# Clinical application of invalid foods using mealworms and evaluation of nutrition status and immune function: a study protocol for a randomized, double blind, placebo-controlled trial

**DOI:** 10.1186/s40795-019-0307-6

**Published:** 2019-11-18

**Authors:** Hyung Sun Kim, Yun Sun Lee, Soo Yun Jang, So Young Jun, Jin Hong Lim, Im Kyung Kim, Hyung Mi Kim, Joon Seong Park

**Affiliations:** 1 Pancreatobiliary Cancer Clinic, Department of Surgery, Gangnam Severance Hospital, Yonsei University College of Medicine, 20, Eonju-ro 63-gil, Gangnam-gu, Seoul, 06229 Korea; 2 Department of Nutrition and Dietetics, Gangnam Severance Hospital, Yonsei University, Seoul, Korea; 3 Department of Surgical critical care medicine, Severance Hospital, Yonsei University, Seoul, Korea

**Keywords:** Nutritional balance, Malnutrition, Immune function, Invalid foods

## Abstract

**Background:**

Protein intake is important for the recovery of the immune system, physical strength, and wound healing after surgery. Sarcopenia is associated with a poor prognosis when compared to patients without sarcopenia in cancer patients. Recently, edible insects, such as mealworms, have been recognized as having a high protein content. In this study, we will evaluate the effect of nutritional status and immune function change based on a patient’s ingestion of mealworms after hepatobiliary pancreatic surgery.

**Methods/design:**

This is a prospective, two-armed, phase III study investigating the effect of mealworm improving nutrition and immune status in patients after hepatobiliary pancreatic surgery. In the trial group, the patients will be provided with mealworms for 2 months after surgery. In the control group, patients will be provided with grain powder for 2 months after surgery. The target for accrual is 168 patients. We divided in to three groups according to the type of surgery.

**Discussion:**

The primary endpoint is to evaluate body cell mass index 2 months postoperatively. Secondary endpoints include other body composition changes as well as nutrition index and immune function change. We expect that ingestion of mealworms can effectively improve the nutritional status and enhance the immune function. Mealworm can be used effectively for nutritional management of patients after surgery.

**Trial registration:**

Clinicaltrials.gov NCT03201926 Registered June 28, 2017, retrospectively registered.

## Background

Approximately 20–50% of hospitalized patients are reported to be malnourished [1]. The nutritional status of a patient generally declines during hospitalization [2, 3]. Poor food intake during hospitalization is caused by deterioration of nutritional status, and malnutrition is often associated with complications including infection, increased number of hospital days, and increased mortality [1, 4, 5]. The American Society of Parenteral and Enteral Nutrition guidelines suggests that patients at risk of malnutrition should be provided with over 80% of their calorie and protein requirements within 2 to 3 days [6]. Patients with sarcopenia imbalance generally have a lower 5-year survival rate in cancer patients [7]. The quality of protein intake is important for the recovery of the immune system, physical strength, and wound healing after surgery; this is problematic since the patient’s protein intake is generally quite poor [8].

In particular, patients who undergo a pancreatectomy or hepatectomy usually have a severe imbalance in their nutritional status due to the disruption of digestive function and nutritional absorption postoperatively. Therefore, it is necessary to use high-quality protein-derived foods that can effectively increase protein intake. The United Nations Food and Agriculture Organization (FAO) is actively encouraging food production of edible insects as a new food source for the future. Currently, various insects are used in food in many regions, including Africa, Asia, South America, and Australia. The edible insects are high in protein content (50–60%) and contain a large amount of fat, fiber, vitamins, and minerals. *Tenebrio molitor* (mealworm), which is an edible insect, contains a large amount of protein and unsaturated fatty acid. The nutritional components of these mealworm are thought to be of value as nutrient sources of high quality in a small amount of patients who need high nutrition.

The purpose of this study is to evaluate the development of long-term personalized products using mealworm and the enhancement of stability, nutritional status and immune status according to ingestion.

## Methods/design

### Study design and period

The trial is a single center, two-armed phase III study. The trial has been registered at www.clinicaltrials.gov (NCT03201926). Patients will be recruited by the Pancreatobiliary Cancer Clinic, Gangnam Severance Hospital, Yonsei University College of Medicine. The expected total duration of patient accrual is 2 years and 6 months and the follow-up period is 6 months.

### Study objectives and endpoints

The primary endpoint is to evaluate body cell mass index at 2 months postoperatively. Secondary endpoints include other body composition changes as well as nutrition index and immune function.

### Patient selection and enrollment criteria

#### Inclusion criteria

- Patients scheduled for surgery with pancreatobiliary disease and liver cancer (HCC, CCC, and metastatic liver cancer)
- Karnofsky performance status ≥70
- Patients who provide informed consent

#### Exclusion criteria

- Patients who underwent palliative surgery
- Patients with uncontrolled preoperative conditions
- Previous history of surgery affecting nutritional status (ex, gastrectomy, colectomy, etc)
- Pregnant and lactating women
- Patients with an allergy to mealworms

### Sample size calculation

The output of the sample size will be based on an independent two-sample t test. It is expected that there will be a difference in body cell mass between the standard meal group (control) and the group ingesting the mealworms. Assuming an alpha value of 0.05 and 1-β (power) of 0.8, 75 samples will need to be obtained for each group; considering the dropout rate of 10%, 84 samples will need to be obtained for each group. The total of the two groups is 168. The primary endpoint will be the number of subjects and body cell mass. A comparison will then be made between the two groups at 2 months. A subgroup analysis of pancreatobiliary and liver disease will also be performed as a secondary endpoint. Patients with pancreatobiliary and liver disease in our clinic have a 2:1 ratio. Therefore, the 168 patients were divided into two groups: one with 112 patients and the other with 56 patients. Patients with pancreaticobiliary disease (Group A & B) and liver (Group C) disease were assigned through stratified randomization. Pancreatobiliary disease group is divided into enteral feeding group(Group A) and non enteral feeding group.(Group B).

### Pretreatment evaluation

All patients who are potential candidates for hepatopancreatobiliary surgery will undergo a standard evaluation that will include contrast-enhanced computed tomography (CT), endoluminal ultrasound (EUS), magnetic resonance imaging (MRI), and positron emission tomography (PET-CT), which will be discussed at the preoperative conference.

Patients recommended for major hepatopancreatobiliary surgery (such as a liver resection, pancreaticoduodenectomy, or total/distal pancreatectomy) will be contacted and provided with a participant information sheet. Patients will be divided into three groups according to the type of surgery.

### Treatment

#### Trial intervention

Patients receiving dietary supplementation with mealworms after their pancreatectomy, bile duct resection and hepatectomy will be given the mealworms during their hospitalization period and for another 2 months after discharge. The patients in group A will undergo enteral feeding after surgery (for those who undergo a pancreaticoduodenectomy, nutrients will be administered by an enteral feeding tube for early feeding while securing the pancreatojejunostomy). A feeding nasojejunal tube will be placed for patients in the enteral feeding group. Enteral feeding (Newcare 300 RTH, Daesang, Korea) will start within 24 h postoperatively at a rate of 20 mL/h. The velocity will be progressively increased by 20 mL/d until reaching the full nutritional goal (25 kcal/kg). Enteral feeding will be delivered by an infusion pump for 18 h/day with 6 h of rest.

On postoperative day 7, abdominal and pelvic CT scans will be obtained, and if there are no complications, we will then start patients on a clear liquid diet for 1 day. In addition, the patients will be permitted to have a full liquid diet for 2 days and then a soft diet for 3 days following the liquid diet.

The patients of group B will undergo oral feeding 2 days after the surgery. (distal pancreatectomy, total pancreatectomy and bile duct resection) We will start patient’s clear liquid diet for 1 day. The patients will be permitted to have a full liquid diet for 2 days and soft diet for 3 days following the liquid diet.

The patients of group C will undergo oral feeding 2 days after surgery (which includes liver resections). We will then start the patients on a clear liquid diet for 1 day. In addition, the patients will be permitted to have a full liquid diet for 2 days and soft diet for 3 days following the liquid diet. The mealworm powder include 3 g of carbohydrate, 14.4 g of protein and 11 g of fat, 163 kcal per 30 g. (Table 1) As a result of a nutritional analysis of mealworm powder, 10.28 g of carbohydrate, 48.26 g of protein and 35.81 g of fat were found per 100 g. In particular, the protein includes essential amino acids as well as many unsaturated fatty acids ranging from 76 to 80% of the total fatty acids. In addition, iron and calcium and other minerals were found (Table 2).

**Table 1:**
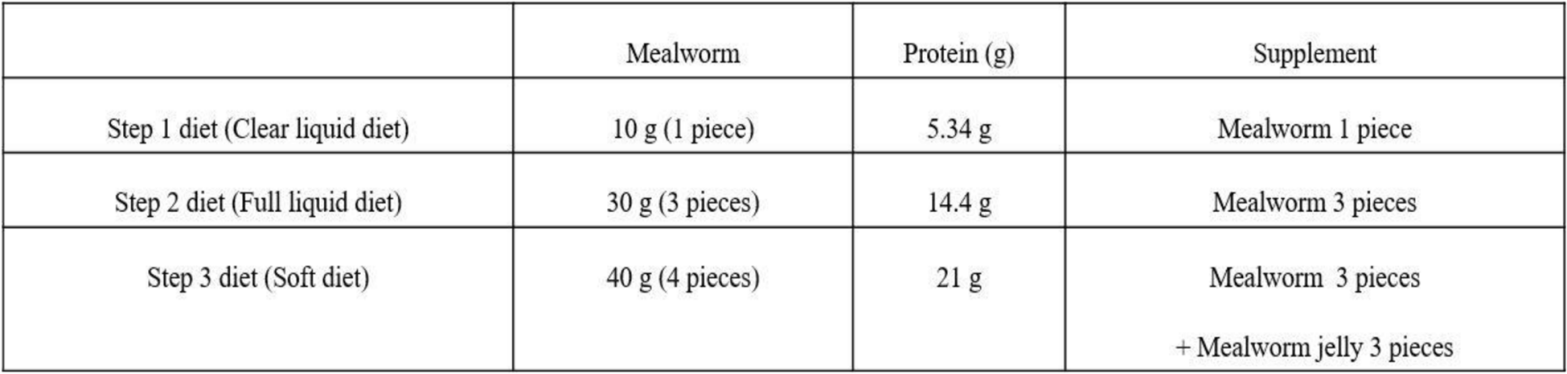
Components of the Trial group’s diet

**Table 2:** Menu plan and protocol of the hospital meals for the mealworm and control groups

			Mealworm group	Control group
Step 1	Menu Nutrient	Energy Protein Carbohydrate Fat	Carbohydrate-based liquid diet, Mealworm jelly 580 kcal 13 g 114 g 8 g	Carbohydrate-based liquid diet, Juice 450 kcal 16 g 84 g 6 g
Step 2	Menu Nutrient	Energy Protein Carbohydrate Fat	Mealworm soup, Mealworm shake with berry, Protein-fortified gelatin 1220 kcal 50 g 180 g 34 g	Carbohydrate-based liquid diet, Thin low-fat soup, Soybean milk, Protein-fortified gelatin 1240 kcal 43 g 180 g 39 g
Step 3	Menu Nutrient	Energy Protein Carbohydrate Fat	Deluxe rice porridge with soft side dishes, ONS1)-based mealworm drink, Mealworm tea-confectionery 1629 kcal 81 g 225 g 45 g	Rice porridge with soft side dishes 1600 kcal 75 g 235 g 40 g
Protocol			The doctor prescribes Step 1 diet (clear liquid diet) as the first meal after surgery. Patients eat this diet for 2–3 meals. As the next meal, the doctor prescribes Step 2 diet (full liquid diet). Patients eat this diet for 2–3 meals. If the diet transition goes smoothly, the doctor prescribes Step 3 diet (soft diet). Patients eat this diet until discharge. The doctor prescribes hospital meal intake. Depending on the patient’s condition, the steps for consuming the diet could be adjusted. The dietitian surveys the dietary intake every day until discharge. If there is a meal-related side effect, it is reported to the doctor.	

#### Control intervention

Patients receiving dietary supplementation with grainpowder after pancreatectomy and hepatectomy will be given grainpowder for their hospitalization period and 2 months after discharge. The grainpowder include 23 g of carbohydrate, 2.9 g of protein and 0.5 g of fat, 106 kcal per 30 g. Group A will receive the grainpowder on postoperative day 7. And group B and C will receive grainpowder in the same dose as trial group’s mealworms at the start of diet at postoperative 2 days (Table 3).

**Table 3:**
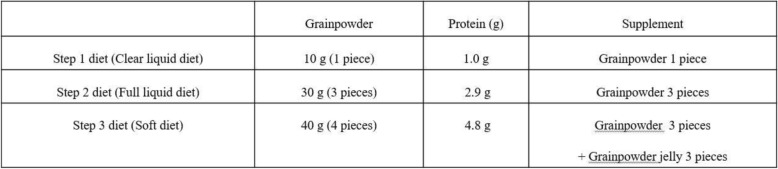
Components of the Control group’s diet

#### Outcome measures

The primary outcome is the body cell mass index as measured by body composition (Inbody S-10 (Biospace, Seoul, Korea)). The secondary outcome measures are as follows: nutritional index (weight, soft lean mass, fat free mass, fat mass, PG-SGA [Scored Patient-Generated Subjective Global Assessment]) and immune function test (that assesses T cells, B cells, cytokines). We will confirm changes in immune cells through FACS (fluorescence-activated cell sorting). Based on the immune assay using blood samples from patients, we will identify changes in several cytokines.

### Timeline and recruitment

#### Recruitment of study participants

Once the patients’ surgery is confirmed, the research staff will give the study staff a list of the patients. After determining the suitability of the patient, the study staff will assign the patient to the test or control group in a randomized sequence in the order in which they are enrolled. In addition, they will be assigned in a double-blinded manner.

#### Randomization

The researchers will select patients after obtaining consent. Randomization will take place via an allocation randomization system 2 days before the surgery, which will be directed by our department. Patients will be randomized to one of the treatment groups in a 1:1 ratio. After consent for study enrollment is obtained, the randomization process will be applied to identify the next allotment. The surgeons will be blind to the allotment throughout the enrollment process.

#### Blinding

Patients and all team members will be blinded to the intervention. Adverse event (AE) and serious adverse event (SAE) need to be reported as soon as they are noted. This will not have any impact on the endpoint assessment of the patient.

#### Data management

The amount of food consumed by the patient daily will be assessed as a diary entry. The researcher collects the bags left over from the patients at the outpatient clinic. Individual participant medical information obtained as a result of this study is considered confidential and disclosure to third parties is prohibited. Blood samples transferred to the laboratory of Gangnam Severance Hospital will be identifiable by unique trial numbers only. Results related to nutrient indicators (Inbody, PG-SGA) will be managed by a Gangnam Severance Nutrition Team. The blood sample associated with the immune function test will be transferred to the lab of the study staff in Gangnam Severance Hospital, which will then undergo FACS (Fluorescence-activated cell sorting).

#### Withdrawal and reporting of adverse effects

If patients wish to voluntarily withdraw from the study, the patient will be asked if they would be given medical care until any AE symptoms resolve or the patient’s condition becomes stable.

Patients can quit the trial at any point without the need to give reasons for their decisions. If voluntary withdrawal occurs prior to diet intervention, the patient will not be randomized, and no more trial data will be collected for that patient. Patients can also withdraw from the trial following diet intervention.

#### Statistical methodology

All data will be entered into a single Microsoft Excel spreadsheet with participants identified only by their unique subject number. All entries will be double-checked by another member of the research team. Statistical analysis will be performed using IBM SPSS version 21.0 (IBM Corp; released 2012. IBM SPSS Statistics for Windows Version 21.0. Armonk, NY, USA). The mean difference in nutrient intakes, changes in body measurements, changes in body composition, and PG-SGA scores will be calculated using the Wilcoxon signed rank test and the Mann Whitney test.

#### Monitoring and follow-up period

The research team is responsible for monitoring the study. At 1 week after discharge, the participants are asked whether they are willing to continue ingesting the mealworm. Every 2 weeks after the patients visit their outpatient clinic, the monitoring researcher will check whether they are taking the mealworm regularly. The research team is also responsible for systematically reviewing the causes of withdrawal from the study. Assessment of these changes will be determined 2 months after surgery (Table 4).

**Table 4:** Clinical trial timeline

		Study period				
		Enrolment	Allocation	Postoperative day		
Timepoint		Day − 2	Day − 1	1	7	60
Enrolment						
Initial eligibility screening		◆				
Informed consent		◆				
Allocation			◆			
Interventions	Mealworm diet			◆	◆	◆
	Control diet			◆	◆	
Assessments 1)Anthropometry 2)Nutrition index 3)Blood test 4)Immune function evaluation	Height, weight, BMI Body composition PG-SGA					
			◆			◆
			◆			◆
	Complete blood count Liver function test Renal function test		◆	◆	◆	◆
	Immune function		◆	◆	◆	◆

#### Ethical and legal considerations

The study protocol was approved by the institutional review board at Gangnam Severance Hospital, Yonsei University of Korea (3–2017-0077). The study complies with the Declaration of Helsinki and the principles of Good Clinical Practice.

## Discussion

The aim of this study is to evaluate the effectiveness and improvement of nutritional status and immune function based on the intake of invalid foods in postoperative patients for major surgery (such surgery of the pancreas, bile duct and liver) after an intervention period of 2 months. Nutritional deficiencies are frequent in patients after abdominal surgery. Malnutrition is an important risk factor for developing sarcopenia [9–12]. The decrease in the amount of fat free mass is associated with an increased mortality rate in patients at risk of malnutrition such as surgery and critical illness [13]. Protein-enhanced diets are known to increase survival in these patients, and therefore, nutritional support using high-protein foods is important [14]. Based on studies such as these, high protein foods using mealworm are now being used. However, only a few studies have evaluated the enhancement of immune function in patients after intake of high protein foods. In other studies, immune function evaluations were reported [15, 16]. Early enteral nutrition after esophageal cancer surgery can effectively improve the nutritional status and enhance the immune function [15] . Some markers of immune function may be useful for distinguishing patients with good or bad prognoses after head and neck cancer surgery by checking the cytokine levels [16] . However, there are no studies on the effects of long term use (2 months) of high protein mealworm that can be used effectively for nutritional management of patients after surgery. This will be the first study to directly identify the relationship between nutritional status and immune function after surgery.

## Data Availability

Data sharing is not applicable to this article as no datasets have been generated or analysed for this study yet.

## Abbreviations

AE: Adverse Event
CT: Computed Tomography
EUS: Endoscopic Ultrasound
FACS: Fluorescence-Activated Cell Sorting
MRI: Magnetic Resonance Imaging
PET-CT: Positron Emission Tomography
PG-SGA: Scored Patient-Generated Subjective Global Assessment
SAE: Serious Adverse Event
